# Pre-emptive Breeding Against Karnal Bunt Infection in Common Wheat: Combining Genomic and Agronomic Information to Identify Suitable Parents

**DOI:** 10.3389/fpls.2021.675859

**Published:** 2021-07-29

**Authors:** Livinus Emebiri, Shane Hildebrand, Mui-Keng Tan, Philomin Juliana, Pawan K. Singh, Guillermo Fuentes-Davila, Ravi P. Singh

**Affiliations:** ^1^NSW Department of Primary Industries, Wagga Wagga Agricultural Institute, Wagga Wagga, NSW, Australia; ^2^Graham Centre for Agricultural Innovation (NSW Department of Primary Industries and Charles Sturt University), Wagga Wagga, NSW, Australia; ^3^NSW Department of Primary Industries, Menangle, NSW, Australia; ^4^International Maize and Wheat Improvement Center, Mexico City, Mexico; ^5^Instituto Nacional de Investigaciones Forestales, Agricolas y Pecuarias, Cd. Obregón, Mexico

**Keywords:** Karnal bunt resistance, *Tilletia indica*, wheat, *Triticum aestivum*, genome-wide association study, GWAS, genomic prediction, grain yield

## Abstract

Wheat (*Triticum aestivum* L.) is the most widely grown cereal crop in the world and is staple food to half the world’s population. The current world population is expected to reach 9.8 billion people by 2050, but food production is not expected to keep pace with demand in developing countries. Significant opportunities exist for traditional grain exporters to produce and export greater amounts of wheat to fill the gap. Karnal bunt, however, is a major threat, due to its use as a non-tariff trade barrier by several wheat-importing countries. The cultivation of resistant varieties remains the most cost-effective approach to manage the disease, but in countries that are free of the disease, genetic improvement is difficult due to quarantine restrictions. Here we report a study on pre-emptive breeding designed to identify linked molecular markers, evaluate the prospects of genomic selection as a tool, and prioritise wheat genotypes suitable for use as parents. In a genome-wide association (GWAS) study, we identified six DArTseq markers significantly linked to Karnal bunt resistance, which explained between 7.6 and 29.5% of the observed phenotypic variation. The accuracy of genomic prediction was estimated to vary between 0.53 and 0.56, depending on whether it is based solely on the identified Quantitative trait loci (QTL) markers or the use of genome-wide markers. As genotypes used as parents would be required to possess good yield and phenology, further research was conducted to assess the agronomic value of Karnal bunt resistant germplasm from the International Maize and Wheat Improvement Center (CIMMYT). We identified an ideal genotype, ZVS13_385, which possessed similar agronomic attributes to the highly successful Australian wheat variety, Mace. It is phenotypically resistant to Karnal bunt infection (<1% infection) and carried all the favourable alleles detected for resistance in this study. The identification of a genotype combining Karnal bunt resistance with adaptive agronomic traits overcomes the concerns of breeders regarding yield penalty in the absence of the disease.

## Introduction

Wheat (*Triticum aestivum* L.) is the most widely grown cereal crop on the planet, a staple of the world economy, supplying one fifth of calories consumed by people each day (Anonymous, 2020). The current world population of about 7.7 billion is expected to increase and reach 9.8 billion people by 2050. To accommodate the increased demand for food, annual cereal production will need to rise by about 60–70% from the current level of 2.8 billion tonnes. For various reasons, however, production is not expected to keep pace with demand in developing countries, and their net imports of cereals are projected to more than double from 135 million metric tonnes in 2008/2009 to 300 million metric tonnes in 2050 (Food Agriculture Organization 2009). This gap can be bridged by increased imports, and significant opportunities exist for traditional grain exporters, including Australia, to produce and export greater amounts of wheat over the next few decades (Linehan et al., 2012). Karnal bunt, a disease caused by the fungus *Tilletia indica* Mitra [syn. *Neovossia indica* (Mitra) Mundkur], is a threat to grain export (Joshi et al., 1983), due to its use as a non-tariff trade barrier by several wheat-importing countries (Beattie and Biggerstaff, 1999). The disease has minimal impact on wheat grain yield (Warham, 1986; Murray and Brennan, 1998) but the infected grains exude an unpleasant, rotten fish odour due to a chemical (trimethylamine) produced by the fungal spores (Mitra, 1935). Trimethylamine is associated with multiple diseases in humans, including renal disorders, cancer, obesity, and cardiovascular diseases (Chhibber-Goel et al., 2016).

Control of this disease is difficult because teliospores of the fungus are resistant to physical and chemical factors (Fuentes-Dávila et al., 2018), the fungus causes local infections (Fuentes-Davila, 1996), and teliospores may remain dormant for more than 32 months (Babadoost et al., 2004). The cultivation of resistant varieties remains the most cost-effective approach to manage the threat of incursions into countries free of the disease (Singh et al., 2007; Emebiri et al., 2019a). Sources of resistance have been identified in the wild relative of wheat, *Aegilops tauschii* (Chhuneja et al., 2008), and in synthetic hexaploid wheat (Mujeeb-Kazi et al., 2006), but resistance in common wheat is limited (Fuentes-Dávila and Rajaram, 1994), and as such, progress in breeding resistant varieties has remained modest. In most wheat-exporting countries that are free of the disease, there are no breeding efforts due to cost burdens and the low return on investments, which in the absence of an incursion, is zero (White et al., 2016). Availability of molecular markers closely linked to resistance genes could be incentivising, as it has the potential to improve selection (Singh et al., 2012; Emebiri et al., 2019b), but efforts in the past have also been modest. Quantitative trait loci (QTL) associated with Karnal bunt resistance in common wheat have been identified in the past (Nelson et al., 1998; Singh et al., 2003; Singh et al., 2007, 2012; Kumar et al., 2007, 2015; Kaur et al., 2016), but these studies were based on a small number of restriction fragment length polymorphisms (RFLP) and PCR-based simple sequence repeats (SSRs). Recently, the use of high-density single nucleotide polymorphism (SNP) arrays in genome-wide association studies (GWAS) have been reported (Brar et al., 2018; Emebiri et al., 2019b; Gupta et al., 2019; Singh et al., 2020), which offers new opportunities for marker-assisted selection (MAS). However, the focus of many plant breeders has now shifted from the use of MAS to the application of genomic selection.

Genomic selection, first introduced by Meuwissen et al. (2001), would be an attractive tool for pre-emptive breeding against exotic pathogens, as it would reduce the challenges of phenotyping (Poland and Rutkoski, 2016). Genomic selection is a two-stage process in which whole-genome markers are used to predict genomic estimated breeding value (GEBV) of individuals in a population, and then selection decisions are made on the basis of these GEBVs (Meuwissen et al., 2001). In the best-case scenario, breeders can select the best performing genotypes from the population for use in their crossing block, without the need to phenotype the plants themselves. The potential for genomic selection has yet to be evaluated for Karnal bunt resistance in common wheat. The prediction accuracy depends on the trait’s heritability, and for Karnal bunt resistance, the estimates are quite high (ranging from 0.75 to 0.91) (Brar et al., 2018; Emebiri et al., 2019a; Gupta et al., 2019; Singh et al., 2020) due to the well-established protocol for disease screening (Fuentes-Dávila et al., 1995).

The International Maize and Wheat Improvement Center (CIMMYT), Mexico, develops novel common wheat germplasm carrying Karnal bunt resistance genes (Singh et al., 2016). Some of the lines were derived from crosses that include Munal#1 (now released as Super 172) and synthetic hexaploids (Mujeeb-Kazi et al., 2006) as parents, and some were developed from backcrosses to commercial varieties, such as Batavia, and Pastor. The lines are important pre-emptive breeding tools to prevent the spread of this quarantined disease into countries that are currently disease-free. However, many other variables are involved in grower uptake of new varieties, with grain yield as the ultimate determinant of which variety the farmer will grow in any given season. In the absence of a disease pressure, genetic resistance may in fact become a liability (yield penalty), as demonstrated in numerous studies (Brown, 2002; Ning et al., 2017). Sharp et al. (2002), for instance, observed that while the *Wsm1* gene in wheat provided the most effective resistance to wheat streak mosaic virus, a mean yield reduction of 21% occurred in the absence of the virus. The wheat stem rust resistance gene, *Sr26*, has a 9% yield penalty (Brown, 2002), and the barley (*Hordeum vulgare*) *mlo* resistance gene has a 4.2% yield penalty (Jorgensen, 1992). This is because genetic resistance is an on-going process, and plants expend metabolic energy that might otherwise be converted to yield. In the absence of the pathogen, existence of a yield penalty for Karnal bunt resistance will outweight the value of the resistance gene (Oliver et al., 2014; Ning et al., 2017), and breeders will be further discouraged from adopting and using improved germplasm in their programmes for fear of upsetting the established phenology and yield profiles.

The key to pre-emptive breeding would be to provide breeders with a package of molecular markers and resistance genes in genetic backgrounds that will not upset established yield and phenology profiles, as there is no point selecting less susceptible varieties if there is an opportunity cost of lower yield without disease. In this paper, we report a research on pre-emptive breeding for Karnal bunt resistance designed to identify linked molecular markers, assess prospects of genomic selection as a tool, and prioritise wheat genotypes suitable for use as parents. To identify such genotypes, we performed field experiments over two years to compare their agronomic values with those of reference, commercial varieties.

## Materials and Methods

### Plant Materials

The germplasm materials consisted of 242 genotypes, made up of 177 bread wheat varieties, 8 durum wheat, 11 triticale, and 46 Karnal bunt-resistant germplasm lines (KBRL). The KBRL were developed at the CIMMYT, and imported into Australia through the CIMMYT-Australia-ICARDA Germplasm Evaluation (CAIGE) suite of projects.^1^ The wheat varieties represent parents used in breeding programmes, historical varieties, and current commercial varieties that are still being cultivated. These were mainly bred in Australia, but some originated from the United States, Brazil, Canada, China, Mexico, New Zealand, and India, providing a global resource for genetic analysis. The bread wheat lines include Super172 (synonym Munal-#1), used as the resistant check, and the highly susceptible Indian wheat variety WL-711 (synonym WL-711-0IND) used as a susceptible check. The names of the varieties, year of release and pedigrees are listed in Supplememntary Table 1.

### Disease Phenotyping

Phenotypic data on Karnal bunt resistance collected from Australian wheat varieties and CIMMYT advanced breeding lines were used. The data for the Australian varieties were derived from field experiments (Emebiri et al., 2019a) conducted during three consecutive cropping seasons (2014–2015, 2015–2016, and 2016–2017), at the Norman E. Borlaug Experimental Station, the CIMMYT, Obregon. The data on CIMMYT breeding lines, collected over three planting dates, were kindly provided by Dr. Ravi P. Singh as part of the materials delivered through CAIGE project. In these data, Karnal bunt resistance was calculated as the percentage of infected grains in each ear (Fuentes-Dávila and Rajaram, 1994), but to rate the genotypes consistently across data sets, those with infection levels of 0–2.5% were rated as resistant, 2.6–5% as moderately resistant, 5.1–10% as moderately susceptible and greater than 10% as susceptible (Gaudet et al., 2001).

### Genotyping

Genomic DNA was isolated from the leaves of individual lines as described in Tan et al. (2015) and genotyped using DArT-Seq technology (Diversity Arrays Technology Pty Ltd., Australia). The polymorphisms were scored as binary data (0/1), indicating the presence/absence of SNP in the genome of each sample. The DArTseq data were filtered for quality, first by removing duplicates and monomorphic markers; then by retaining markers on the basis of CallRate (≥0.95), reproducibility (≥0.95), minor allele frequency (≥0.05), and percent missing data (≤15%). The final molecular marker data set comprised of 8,012 loci scored on 177 hexaploid genotypes. All heterozygotes were treated as missing data, and the corresponding values were imputed using the Random Forest regression method in R package (Stekhoven and Bühlmann, 2012).

### Genetic Structure and Linkage Disequlibrium

Genetic structure was analysed using algorithms implemented in the *adegenet* package (Jombart, 2008). First, we ran the *snapclust* function to select the optimal number of genetic groups, based on a statistical measure of goodness of fit, the Bayesian Information Criterion (BIC). Then, a discriminant analysis of principal components (DAPC) was applied, which combined PCA with discriminant analysis to maximise between-group differences while minimising the within-group variation (Jombart et al., 2010).

Linkage disequilibrium (LD) (statistical association between allelic variants) was calculated in plink v1.9 (Purcell et al., 2007) as the squared correlation coefficient (*r*^2^) between alleles at pairs of loci within each chromosome. The analyses were carried out with a molecular data set that was thinned down evenly across the genome to a window size of 8 kb. The decay of LD over genetic distance was examined by plotting the pair-wise LD against distance, and fitting a decay curve, established by square root transformation of the predicted LD values calculated according to Andreescu et al. (2007). The background *r*^2^ value was calculated as the 95^*th*^ percentile of all LD values between markers located on different chromosomes, assumed to unlinked (Breseghello and Sorrells, 2006).

### Genome-Wide Association Analysis

Genome-wide analyses were performed with the R package, *lmem.gwaser* (Gutierrez et al., 2016), according to the Kinship model, which had a lambda value of 1.03. It can be described as follow:

y=xβ+zu+ε,

where *y* is the observed phenotype, *x* is the molecular marker score matrix, β is the vector of marker allelic effects, *z* is an incidence matrix, *u* is a vector of random polygene background effects with Var(u) being 2KV_*G*_ (K = Kinship coefficients and V_*G*_ = genetic variance), and ε is a vector of random experimental error.

We adjusted observed *P*-value for multiple testing using two methods: the method of Li and Ji (2005), which is based on the effective number of independent tests (alpha level of 0.05) and the false discovery rate (FDR) method of Benjamini and Hochberg (1995). The method of Li and Ji (2005) was implemented in the *lmem.gwaser* package but FDR was calculated in the R function, *p.adjust()*. Allelic effects and proportion of phenotypic variance (*R*^2^-values) explained by significant markers were derived from simple linear regression analyses, with *R*^2^ = SS_*reg*_/SS_*tot*_, where SS_*reg*_ is the regression sum of squares and SS_*tot*_ is the total sum of squares.

### Physical Mapping

Significant markers were assigned to physical positions in megabase pairs (Mbp) by nucleotide BLAST (BLASTN) search (E-value threshold = 1E-5) against the IWGSC RefSeq v1.0 Chinese Spring assembly,^2^ using the marker sequence for query. High-confidence candidate genes closely matching the marker sequence were obtained in a window size of estimated LD each side of the marker. The results were further refined with the *JBrowse* tool (Buels et al., 2016) to identify nearby wheat expressed sequence tags (wEST), and this allowed assigning the markers to physical bin positions on the deletion maps of the Chinese Spring cultivar.

### Prediction of Karnal Bunt Resistance

Two scenarios were considered for genomic prediction: (1) the use of only the markers identified in GWAS analysis (QGBLUP), analogous to marker-assisted selection strategy and (2) the use of genome-wide markers to predict the performance of individuals for which genotypic data is available, but not the phenotypes. The analysis was carried out using the genomic best linear unbiased prediction (GBLUP) model (Meuwissen et al., 2001), in which the G-matrix was calculated using either the six significant markers identified in GWAS, or the genome-wide markers, depending on the approach. In both cases, accuracies were determined from a fivefold cross-validation scheme, in which 80% of the genotypes were randomly assigned to a training set (TRN) and the remaining 20% to a testing set (TSN). This was repeated 100 times, and for each repeat, the individuals in the TRN and TSN set were randomly re-sampled, the phenotypes of individuals in the TST set were masked, and then predicted based on the TRN set. Genomic prediction accuracy was calculated as the Pearson’s correlation between the actual and the predicted phenotypes of the lines in the TSN set.

### Agronomic Assessment

Field experiments were carried out in 2015 and 2016 cropping seasons to assess the agronomic value of Karnal bunt-resistant lines. For this study, 37 of the Karnal bunt resistant lines from CIMMYT were used. Seven commercial wheat varieties were included as reference genotypes. These included Super172 (syn. Munal#1), Axe, Mace, Rosella, Scout, Suntop, and Waagan. Axe was released in 2007 and is a very early maturing wheat that is suited for short growing seasons, while Mace, released in 2008, has broad adaptation, with consistently high yield under a wide range of conditions. Rosella is a widely adapted winter wheat used for dual-purpose grazing, while Suntop was released in 2011 as a main season line, with high and stable yields from low to high yield potential areas. Both Scout and Waagan were released in 2009. Scout is a mid-season maturity variety with low screenings and high test weight, and Waagan is a very early maturing spring wheat, with high yield potential in medium/low rainfall environments.

The experiments were conducted at the Wagga Wagga Agricultural Institute, Wagga Wagga NSW, Australia (latitude –35.05° S, longitude 147.35° E), on a site with well-drained, sandy clay loam soil with a greyish brown colour. The experiments were arrayed in a row-column, *p*-rep design (Cullis Brian et al., 2006), with experimental units (plots) measuring 7.5 m^2^ in area (six rows with 30 cm spacing, 6 m long, trimmed to 5 m prior to harvest). Plots were sown with a tractor-mounted Seeder, at a rate of 60-g seeds per plot. All experiments were fertilised at the time of sowing with monoammonium phosphate at the rate of 100 kg/ha, and standard operational procedures (irrigation, weed, pest/disease control) were applied.

### Statistical Analysis of Agronomic Data

Data on the following agronomic traits were collected: emergence counts (number of plants per plot), flowering date (50% awn emergence), plant height (height from soil to tip of the awns), NDVI (at anthesis using the GreenSeeker) and grain yield (weight of the uncleaned seed weight from machine harvests per plot). At harvest, the uncleaned grains (300 g) were subsampled and used to collect data on grain size (1,000 grain weight) and grain plumpness (grains retained over a 2.5 mm sieve).

A two stage approach was used for data analysis. In the first stage, each trait within an experiment/year was analysed separately to account for design factors and spatial field variation. This was performed using a mixed linear model framework with spatial corrections for field heterogeneity as implemented in the R package, *SpATS* (Rodríguez-Álvarez et al., 2018). The analytical model included data on seedling emergence (count) per plot as a fixed component to adjust for differences in plant density. *SpATS* uses two-dimensional smoothing surfaces with penalised splines to model the spatial trends within the field and obtain estimates of predicted means. In the second stage, adjusted means for the 2 years were jointly modelled to generate variance components, and a genotype × trait matrix, which was analysed according to the genotype plus genotype-by-environment method, as implemented in *GGEBiplotGUI* (Frutos et al., 2014). Graphical displays of the output were aided by the R package, *ggplot2* (Ginestet, 2011).

## Results

### Phenotypic Variation

Broad-sense heritability of Karnal bunt resistance, calculated as the ratio of genotypic to phenotypic variance components, was 0.83 ± 0.02, and for narrow-sense heritability, calculated using a marker-based approach (Covarrubias-Pazaran, 2016), the estimate was relatively high at 0.61 ± 0.14. These estimates indicated a large contribution of genetic factors to Karnal bunt resistance in the wheat accessions. The average percentage infection in the wheat accessions was 17.5%, with a range of 0.4–51.8%. There were 10 resistant lines, that is, genotypes with seed infection levels of 0–2.5%. These included seven KBRL and three cultivated varieties. Sixteen of the accessions were moderately resistant (% KB infection > 2.5–5%), 46 were moderately susceptible (>5–10%) and 105 were susceptible (%KB infection > 10%).

### Genetic Structure

**FIGURE 1:**
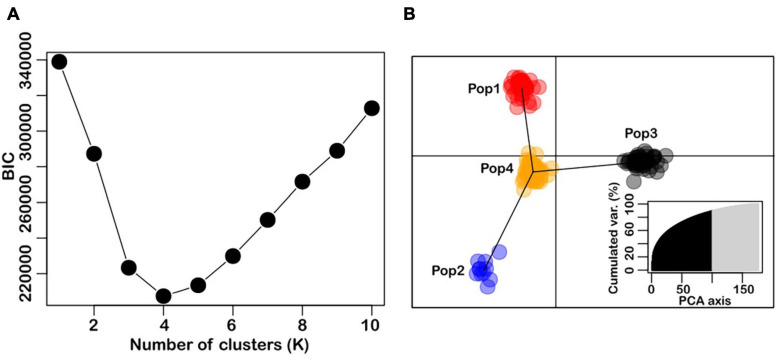
Population structure in the panel of 177 wheat accessions used for the study. Panel **(A)** is the optimal number of clusters identified with the find.cluster function in adegenet (Jombart, 2008). Panel **(B)** is the DAPC results, showing relative positions of individuals and genetic clusters in the discriminant space (inset is the PCA eigenvalues).

Genetic structure analysis was performed to determine whether the composition of wheat accessions was structured, that is, differentiated into clusters of closely related individuals, and which individuals belong to which clusters. Graph of BIC values showed a minimum value at K = 4, and this was determined to be the optimal number of genetic clusters in the wheat accessions (Figure 1A). The DAPC analysis showed clear separation of the accessions into four genetic clusters (Figure 1B), with sample sizes ranging from 11 to 79. It was noteworthy that the CIMMYT-derived Karnal bunt resistant lines cluster together in Pop3 (n = 46), and along with varieties such as Seri-M82, Pastor, Genaro-F81, and Veery5, they were separate from the other wheat genotypes. Seri-M82 and Genaro-F81 are semi-dwarf, historical wheat varieties from CIMMYT, and Pastor is derived from a cross involving Ser-M82 as a parent. The genotypes in Pop1 (*n* = 41) and Pop2 (*n* = 11) were Australian-bred wheat varieties, and those in Pop4 (*N* = 79) were a mixture of global wheat genotypes. They include Australian varieties such as Axe, Drysdale and EGA-Burke, the Indian variety WL-711, Canadian varieties such as AC-Domain and its progeny, AC-Snowbird, the Chinese variety, Chuan-Mai-18, the Brazilian variety, Carazinho and the USA variety, Angus.

**FIGURE 2:**
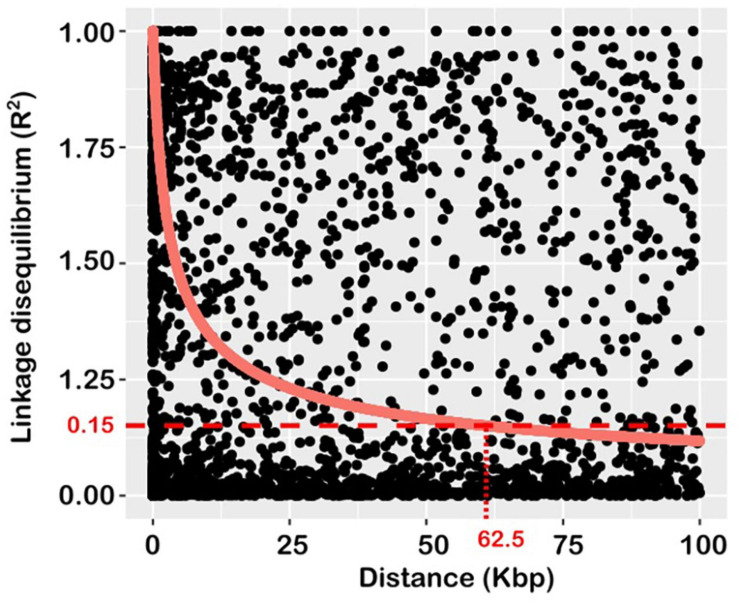
Plot of average linkage disequilibrium (LD) values (r2) against inter-marker distances over a short (100 kbp) distance to visualise LD decay. The decay curve is the square root transformation of predicted LD values according to Andreescu et al. (2007). The horizontal and vertical dotted lines indicate the baseline r2 threshold value, and the extent of LD decay, respectively.

Linkage disequilibrium (statistical association between allelic variants) and its decay rate were examined using pair-wise combinations of markers genotyped across the 21 wheat chromosomes. The estimate of background LD, calculated from *r*^2^ values of unlinked markers was 0.15, which agrees with the value commonly reported for wheat (Joukhadar et al., 2020). This value intersected the LD decay line at 62.5 Kbp (Figure 2), and this represents the extent of LD in the population used for this study. It represents the mapping resolution of any QTL detected and was used as the confidence interval.

### QTL Identification

There was an evident association between genetic groups and Karnal bunt resistance in the population, as majority of the lines in Pop3 were resistant, and separate from the other groups in the DAPC space (Figure 1B). This association of population group with resistance was statistically significant, as determined from a chi-square test of independence (*X*^2^ = 54.81, *P*-value < 0.001), hence, corrective measures were applied to adjust for the potential bias in declaring QTL identification.

**FIGURE 3:**
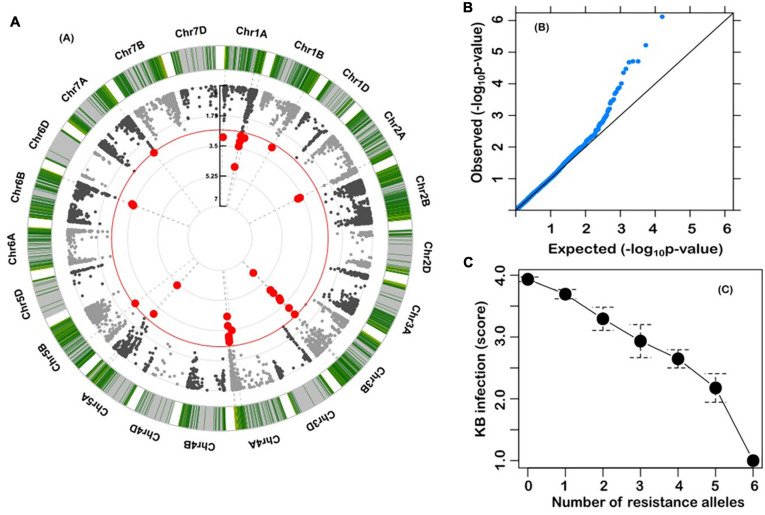
**(A)** Circular Manhattan plot from genome-wide scan with a mixed linear model. The red line is the significance threshold; **(B)** QQ plots from genome-wide scan. The late separation between observed and expected P-values in the upper left section represents the significant associations; and **(C)** Relationship between number of favourable alleles and Karnal bunt resistance in the wheat accessions.

A Kinship-corrected GWAS analysis identified six markers that were significantly associated with Karnal bunt resistance, after controlling for multiple testing using both the genome-wide threshold and FDR criteria (Table 1 and Figure 3A). We compared different mixed models and found the kinship model as the most effective to correct for population structure, as it produced the lowest genomic inflation factor (lambda, λgc = 1.03), and the observed *P*-values showed little deviations from the expected (Figure 3B). Surprisingly, all the significant markers were in the A and B genomes, and physically localised to the long arms of chromosomes 1A, 2A, 3B, 4A, 5A, and 6B (Table 1). The markers explained a large proportion (7.6–29.5%) of the variation in Karnal bunt resistance, and when favourable alleles were considered, genotypes with a high number of beneficial alleles were completely resistant to Karnal bunt infection (Figure 3C).

**TABLE 1:** Summary of significant markers detected in association mapping of Karnal bunt resistance in common wheat.

**Peak marker**	**Chr.**	**Physical position (Mb)**	**Deletion bin**	***P*-value**	**FDR-value**	**Allelic effect**	**R-squared (%)**
2282741	1A	481.52	1AL1-0.17-0.61	2.08E-05	3.33E-02	1.10	26.91
1249729	2A	723.62	C-2AL1-0.85	1.45E-04	1.16E-01	0.92	7.58
1037716	3B	618.02	3BL10-0.50-0.63	7.64E-07	6.12E-03	-1.04	29.47
993727	4A	719.43	4AL4-0.80-1.00	4.44E-05	5.08E-02	-1.03	26.31
1128414	5A	618.27	5AL17-0.78-0.87	6.07E-06	2.43E-02	-1.06	27.87
989877	6B	683.23	6BL5-0.40-1.00	3.06E-04	1.88E-01	0.26	8.09

*Nominal *P*-values were adjusted using the false discovery rate (FDR) method of Benjamini and Hochberg (1995); the explained variation (R-squared) and allelic effects attributable to each marker were derived from simple linear regression analyses.*

### Genomic Prediction

**FIGURE 4:**
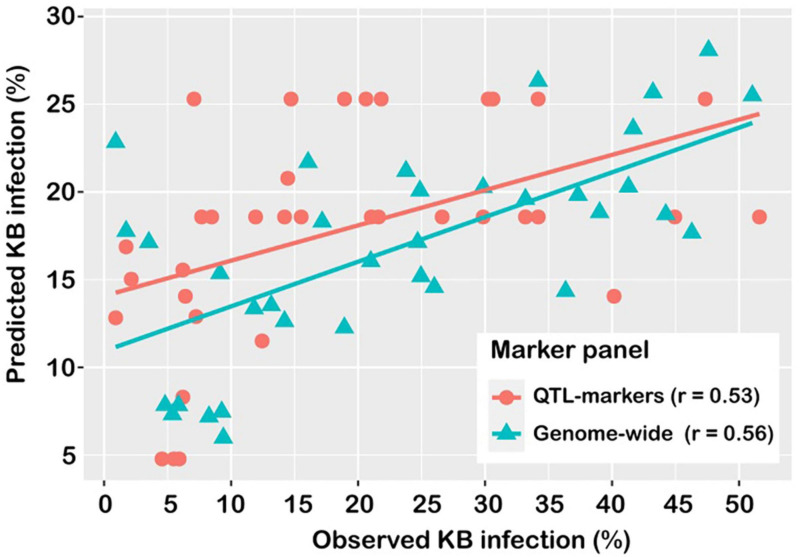
Prediction accuracy of models for Karnal bunt resistance, using markers detected in GWAS analysis (QTL), and genome-wide markers (WG).

There was no difference in prediction accuracy between the QGBLUP approach and the whole-genome prediction (GBLUP) approach (Figure 4). In the QGBLUP approach, the prediction ability for Karnal bunt resistance averaged 0.53 ± 0.003, and in the alternate approach of whole genome marker prediction, the accuracy averaged 0.56 ± 0.01. In effect, genomic prediction using a few, trait-specific markers produced accuracies that compared favourably with those from whole-genome markers.

### Agronomic Profiles

**FIGURE 5:**
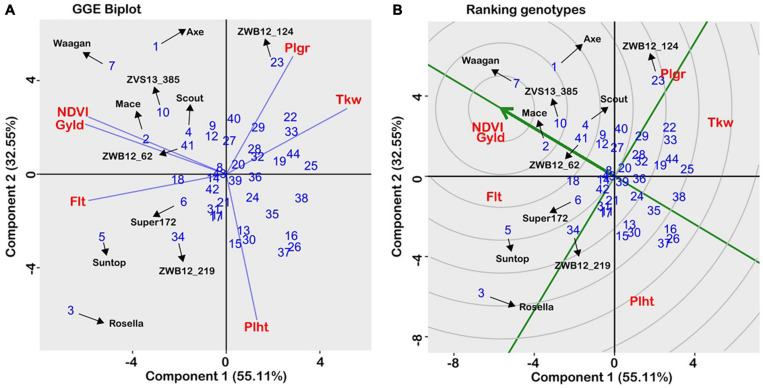
GGE biplot of a genotype × trait matrix averaged over two years, using trait-focussed SVP, and Double-Centred GE with scaling by standard deviation. **(A)** Polygon plot of “which won where.” The numbers refer to individual wheat accessions (see Table 1), and abbreviations are given for trait names. Flt, flowering time; Gyld, Grain yield; NDVI, Normalised difference vegetative index; Tkw, Thousand kernel weight; Plht, Plant height. **(B)** Ranking the accessions in relation to the “ideal” genotype.

In the agronomic experiments, estimates of trait heritability, independent of year and heterogeneous field conditions, were consistently high across traits (Table 2), indicating strong genotypic main effects. The adjusted trait means were analysed using the GGE biplot method to allow visual examination of genotype performance across multiple traits, and identification of superior individuals. The biplot captured 87.7% of total variability in the data (Figure 5) and is therefore appropriate for visualising the relationships among traits. All traits were equally important, as indicated by the relative length of their vectors. The biplot showed that grain yield was positively related to growth duration and biomass production (acute angles), negatively related to plant height (obtuse angle), and independent of grain size (near right angles). When the “which-won-where” function was used to partition the data into a two-dimensional polygon view, the agronomic traits were grouped into three major sectors (Figure 5A): phenology (flowering time/plant height), grain yield (grain yield/NDVI), and grain size (1,000-kernel weight/grain plumpness). Vertex genotypes in each sector are considered the best/worst for traits within the sector (Yan and Rajcan, 2002). Thus, the late-maturing variety, Rosella, was placed at the apex of the phenology sector, while early maturing varieties, Waagan and Axe, were placed at the vertex of the grain yield sector (Figure 5A). These varieties were positioned opposite to the plant height vector, which is consistent with the negative relationship between plant height and grain yield. Of the CIMMYT-derived accessions, ZWB12-124 and ZWB12_147, had the best agronomic values for grain size/plumpness, while ZWB12_158 and ZWB12_30 were the worst for plant height (Figure 5A). The mean trait value for all genotypes are presented in Table 2 to validate the interpretations.

**TABLE 2:** Spatially adjusted means of check varieties and CIMMYT-derived, Karnal bunt resistant germplasm.

**Source**	**Disease rating**	**Grain yield (t ha^–1^)**	**Flowering date (days)**	**Plant height (cm)**	**1,000 Kernel weight (g)**	**NDVI**	**Plump grains (%)**
σ^2^genetic		1.14	22.51	68.44	42.47	0.00	17.96
σ^2^residual		0.14	4.18	12.56	2.84	0.00	5.343
Heritability		0.94	0.92	0.92	0.97	0.87	0.87
SE heritability		0.08	0.11	0.11	0.04	0.16	0.16
Adjusted genotype means							
Axe	S	4.75	128.64	85.72	43.90	0.57	91.83
Mace	S	5.11	128.75	91.28	41.90	0.57	89.83
Rosella	S	5.04	135.14	97.26	38.02	0.51	85.17
Scout	S	5.56	129.55	92.28	44.76	0.58	91.72
Suntop	S	5.26	130.28	94.08	41.78	0.60	84.34
Super172	R	4.92	130.34	94.66	45.11	0.59	89.02
Waagan	S	5.45	128.54	86.36	41.06	0.57	90.39
ZVS13_312	MR	5.35	130.31	93.75	47.87	0.59	91.02
ZVS13_316	MS	4.84	129.95	91.07	47.44	0.61	91.68
ZVS13_385	R	4.69	128.34	89.51	43.93	0.57	89.94
ZVS13_404	MS	5.15	129.13	97.73	44.57	0.60	90.96
ZVS13_406	MR	4.46	128.54	89.66	49.69	0.59	88.86
ZVS13_441	MS	4.79	129.29	99.56	44.67	0.61	92.24
ZWB10_44	R	5.09	128.96	94.61	46.44	0.58	90.29
ZWB10_76	MR	5.36	129.40	99.08	47.03	0.60	89.76
ZWB11_153	MS	4.73	129.06	99.98	49.49	0.60	91.83
ZWB11_172	R	5.07	130.97	97.13	45.16	0.57	91.37
ZWB11_95	MR	5.57	130.55	94.10	44.15	0.58	90.54
ZWB12_103	MS	5.33	128.96	94.81	51.79	0.59	91.94
ZWB12_121	MS	4.75	128.68	94.32	47.88	0.60	91.19
ZWB12_122	MS	5.16	129.22	95.93	47.63	0.63	89.61
ZWB12_123	MS	4.71	129.58	92.15	52.55	0.58	93.66
ZWB12_124	MS	4.93	128.26	88.84	52.70	0.57	93.69
ZWB12_14	R	4.97	129.23	96.78	48.14	0.61	91.46
ZWB12_147	MS	4.65	128.43	96.26	51.82	0.61	93.46
ZWB12_158	MS	4.73	128.72	101.65	47.69	0.59	92.88
ZWB12_16	S	5.14	128.40	93.55	46.60	0.58	92.34
ZWB12_168	S	5.13	130.14	93.86	49.10	0.58	93.11
ZWB12_18	MR	4.57	128.84	92.68	49.05	0.58	93.02
ZWB12_187	MR	5.56	130.75	99.05	48.18	0.62	91.03
ZWB12_189	MS	4.82	127.85	97.52	43.74	0.60	90.55
ZWB12_194	MS	4.79	128.53	94.78	48.26	0.59	92.55
ZWB12_202	S	4.89	128.75	94.30	50.25	0.55	94.61
ZWB12_219	MS	4.75	130.45	96.43	44.21	0.52	88.04
ZWB12_24	MS	4.91	129.47	98.90	47.51	0.60	92.98
ZWB12_29	MS	5.31	130.32	95.10	49.92	0.61	91.83
ZWB12_30	R	5.17	129.73	100.39	50.20	0.59	90.88
ZWB12_31	MR	5.32	129.60	98.16	51.10	0.60	93.32
ZWB12_4	MR	5.20	129.48	95.07	48.22	0.59	90.91
ZWB12_42	MS	5.25	128.84	91.86	48.29	0.60	92.47
ZWB12_62	MR	4.80	129.33	91.24	45.91	0.59	90.12
ZWB12_63	MS	4.73	129.11	94.99	46.19	0.58	89.93
ZWB12_67	MS	5.15	129.40	94.55	46.79	0.58	90.96
ZWB12_86	MS	5.27	128.11	95.54	51.17	0.60	93.19
							

The GGE biplot can also be used to visualise genotype ranking against the “ideal.” The “ideal” is defined as a genotype that combines all favourable attributes, and in Figure 5B, the arrow indicates where the ideal genotype should be. Accordingly, the ideal genotype is expected to be high yielding, early maturing and below average in plant height. A performance line passing through the origin is used as a reference, and a genotype closer to the “ideal” is considered more desirable than those further away. As shown in Figure 5B, Waagan, followed by Mace and Axe are the more desirable of the check varieties, while Rosella and Suntop were far from the ideal genotype. Of the Karnal bunt-resistant accessions, ZVS13-385 and ZWB12-62 were the closest to the ideal genotype (Figure 5B). In particular, the genotype ZVS13-385 was placed within the same concentric ring as Mace, which meant it had similar agronomic attributes. This is relevant information, as Mace is one of the most widely grown varieties in Australia. DNA analysis showed that ZVS13-385 possessed all six of the favourable alleles identified for Karnal bunt resistance, and therefore would be suitable as a parent for transferring resistance into commercially acceptable backgrounds.

## Discussion

In the first part of this study, we sought to dissect the genetic basis of Karnal bunt resistance, as the information is essential for confirming resistance sources, identifying those most suitable as donor parental lines, and designing strategies to accelerate transfer of resistance into commercial cultivars. We identified six DArTseq markers, which explained between 7.6 and 29.5% of the observed phenotypic variation and were located at chromosome positions previously reported in the literature (Bishnoi et al., 2020). When BLASTN search was conducted against the IWGSC RefSeq v1.0 Chinese Spring assembly, the most frequently identified putative candidate gene at the QTLs encoded the F-box domain containing proteins. The F-box proteins are a large superfamily that play pivotal roles in host-pathogen interactions through targeting substrates into the degradation machinery (Cao et al., 2008).

The knowledge that Karnal bunt resistance is mediated by multiple genes is supported by previous studies, but this introduces another dimension to the difficulties of breeding for resistance in the absence of the pathogens (Emebiri et al., 2019b). The multi-gene control implies that marker-assisted selection by pyramiding or stacking of favourable alleles may not be successful (Langridge and Waugh, 2019), because interactions among QTL/genes and environmental factors can make substantial contributions to variation in complex traits such as disease susceptibility (Carlborg and Haley, 2004). As suggested in Emebiri et al. (2019b), new and innovative strategies will be required, and in this study, we assessed the potentials of the method of genomic prediction as a pre-emptive breeding tool. For developing the prediction model, we compared the traditional use of whole-genome markers against the use of a few significant markers identified by GWAS and found the prediction abilities to be comparable (Figure 4). This was not surprising, as genomic prediction accuracy is highly dependent on the LD between the genotyped markers and actual causative variants (de Los Campos et al., 2013). The use of significant trait-specific markers was expected to improve genomic prediction, and in fact, prior marker selection has been suggested as a strategy to increase reliability of the genomic estimated breeding values (Brøndum et al., 2015). Rutkoski et al. (2012) reported that in wheat, genomic predictions based on QTL targeted markers for fusarium head blight resistance (deoxynivalenol) alone were higher than predictions based on genome-wide markers. Other researchers have also found higher prediction abilities of the MAS approach over whole-genome prediction (Slavov et al., 2014; Zhao et al., 2014; Boeven et al., 2016), but Gaikpa et al. (2020) found the opposite to be the case. Similarly, while some researchers have found that use of trait-specific markers as fixed factors increased accuracy of genomic prediction (e.g., Daetwyler et al., 2014), others have observed no difference (e.g., Rice and Lipka, 2019). Invariably, this will vary with trait, and the performance of such a prediction model should be explored on a trait-by-trait basis prior to its implementation in a breeding programme (Rice and Lipka, 2019). Karnal bunt resistance in this population showed high heritability (0.83 ± 0.02), hence marker-based prediction accuracies were almost comparable to genome-wide prediction accuracies. This may not be the case in different populations, but the possibility of using a few significant markers for genomic prediction would augur well for pre-emptive breeding against Karnal bunt infection in countries that are free of the disease, where phenotyping would be difficult and the costs for high-density genotyping can be limiting. This is a subject that requires further investigation, as large-scale studies are showing that, in a high LD crop like wheat, high-density genomic coverage has minimal impact on the genomic predictabilities (Juliana et al., 2019).

The identification of parental lines combining Karnal bunt resistance with adaptive agronomic traits is key to pre-emptive breeding, as it addresses breeder’s concerns regarding yield penalty in the absence of the disease. Plant breeders use the GGE biplot technique for prioritising genotypes for use as parents in varietal improvement as the regular stability analysis does not provide information on the relative ranking of entries with reference to an ideal genotype (Yan and Kang, 2003). The current research carried out a comprehensive examination of Karnal bunt resistant germplasm from CIMMYT and has identified an ideal genotype, ZVS13_385 (TAM200/PASTOR//TOBA97/3/HEILO), which showed agronomic similarity to the highly successful Australian wheat variety, Mace (Moffat et al., 2015; Table 2; Figure 5B). Furthermore, ZVS13_385 is phenotypically resistant to Karnal bunt infection (<1% infection), and possessed all favourable alleles detected for major and minor QTL linked to resistance. This means that it could be used directly as a cultivated variety, or as an ideal genotype for use in the crossing block. We conclude that the identification of a genotype combining Karnal bunt resistance with adaptive agronomic traits negates the concerns of breeders regarding yield penalty in the absence of the disease. Using mathematical modelling, Vyska et al. (2016) showed that even when disease outbreak is uncertain, growing resistant varieties is an optimal strategy for crop protection as it reduces the probability of an outbreak occurring. We add that wide availability of Karnal bunt resistant lines may encourage countries to relax the zero-tolerance regulation that currently exists for Karnal bunt, which is quite costly to implement (Babadoost, 2000; Vocke et al., 2010).

## Data Availability Statement

The raw phenotypic data supporting the findings of this article are available and accessible through the CIMMYT-Australia-ICARDA Germplasm Evaluation (CAIGE) suite of related projects (Biosecurity risk – CAIGE (caigeproject.org.au). The genotypic data can be made available by the senior author with no reservations.

## Author Contributions

LE conceived the study and drafted the manuscript. SH conducted the agronomic experiments. M-KT conducted DNA extractions and supervised the genotyping work. PJ assisted with data analyses. PS, GF-D, and RS conducted the Karnal bunt screening experiments. All authors read and revised the manuscript.

## Conflict of Interest

The authors declare that the research was conducted in the absence of any commercial or financial relationships that could be construed as a potential conflict of interest.

## Publisher’s Note

All claims expressed in this article are solely those of the authors and do not necessarily represent those of their affiliated organizations, or those of the publisher, the editors and the reviewers. Any product that may be evaluated in this article, or claim that may be made by its manufacturer, is not guaranteed or endorsed by the publisher.

## Footnotes

1
http://caigeproject.org.au/

2
https://urgi.versailles.inra.fr/blast/
